# Effect of region of interest on ADC and interobserver variability in thyroid nodules

**DOI:** 10.1186/s12880-019-0357-x

**Published:** 2019-07-12

**Authors:** Xiang Zhou, Chao Ma, Zhi Wang, Jia-ling Liu, Yuan-peng Rui, Yue-hua Li, Yi-feng Peng

**Affiliations:** 1 Department of Radiology, Putuo Hospital, Shanghai University of Traditional Chinese Medicine, 164 LanXi Road, Putuo District, Shanghai, 200062 China; 2 Department of Radiology, Changhai Hospital of Shanghai, The Second Military Medical University, Shanghai, 200433 China; 3 Department of Radiology, No.6 People’s Hospital, Shanghai Jiaotong University, Shanghai, 200233 China

**Keywords:** Thyroid nodule, DWI, ADC, Region of interest, MRI

## Abstract

**Background:**

To determine the effect of region of interest (ROI) on tumor’s apparent diffusion coefficient (ADC) and interobserver variability in thyroid nodules.

**Methods:**

Thirty-three individuals with 45 pathologically-confirmed thyroid nodules were assessed by preoperative diffusion-weighted imaging (DWI) with b values of 0 and 400 s/mm^2^, respectively. Two readers evaluated the ADC values of lesions based on three ROI techniques: whole-volume, single-slice and small solid-sample groups. Interobserver variability was analyzed for all ROI techniques, and the mean ADCs of benign and cancerous thyroid nodules were compared.

**Results:**

For the mean ADCs of non-cancerous thyroid nodules, average differences and limits of agreement (LOAs) between readers were 0.00 [− 0.17–0.17] × 10^− 3^ mm^2^/s for whole-volume ROI (ICC = 0.967), 0.00 [− 0.26–0.26] × 10^− 3^ mm^2^/s for single-slice ROI (ICC = 0.932) and − 0.02 [− 0.38–0.41] × 10^− 3^ mm^2^/s for small solid-sample ROI (ICC = 0.823). For the mean ADCs of cancerous thyroid nodules, average differences and LOAs between readers were − 0.05 [− 0.23–0.13] × 10^− 3^ mm^2^/s (ICC = 0.885), 0.01 [− 0.23–0.25] × 10^− 3^ mm^2^/s (ICC = 0.839) and − 0.07 [− 0.52–0.39] × 10^− 3^ mm^2^/s (ICC = 0.579) for the three ROI methods, respectively. The mean ADC values were more scattered in the small solid-sample ROI group in comparison with the whole-volume and single-slice groups, in noncancerous and cancerous specimens. Of all three ROI techniques, whole-volume ROI-determined ADC had the highest combined sensitivity (80.0%), specificity (88.3%) and Youden index (0.683), with a cut-off of 1.84 × 10^− 3^ mm^2^/s.

**Conclusions:**

The ROI method overtly affects ADC measurements in benign and cancerous thyroid nodules. Small solid-sample ROI yielded the worst interobserver variability of average ADC measurements.

## Background

Thyroid nodules are commonly detected in the adult population, and can be diagnosed as noncancerous or cancerous. Diffusion-weighted imaging (DWI) is an emerging technique for evaluating head and neck tumors, representing a non-invasive method for measuring the diffusion of molecular water, which has the potential to distinguish tissue properties and physiological features. Recently, DWI with derived apparent diffusion coefficient (ADC) has been applied to differentiate noncancerous thyroid nodules from cancerous ones quantitatively [1–20]. Because of the heterogeneity of tumor tissues, ADC measurements may depend on region of interest (ROI) selection. In DWI, ROIs obtained by three main ROI techniques, i.e. whole-volume, single-slice, and small solid-sample, have been applied for obtaining the ADC values of tumors [21, 22]. However, it is rarely assessed for thyroid nodules. The current study aimed to evaluate the effect of ROI selection on ADC measurements and interobserver variability in thyroid nodules.

## Methods

### Patients

Forty-five patients with thyroid nodules were recruited between September 2013 and December 2014, with signed informed consent obtained from all participants. The inclusion criteria comprised: diameter of thyroid nodules larger than 6 mm; no motion artifacts; no contraindications for MRI; no history of thyroidectomy or radiotherapy. Eight cases were excluded for thyroid nodules smaller than 6 mm, which makes it hard to identify the border using ADC measurements. Four additional patients with obvious movement artefacts on DWI images were also excluded. Therefore, 33 patients (7 males and 26 females; mean age, 52.2 ± 10.3 years; age range, 25–71 years) with 45 (30 benign and 15 malignant) thyroid nodules confirmed by histopathological findings were finally enrolled in the current study. The histopathological types of the thyroid nodules are listed in Table 1.

**Table 1:** Histopathological types of thyroid nodules in the evaluated patients

Pathological classification	Number of nodules	Number of cases	Age	
			Range	Average
Papillary carcinoma	14	12	37~64	51.5
Follicular carcinoma	1	1	28~28	28.0
Adenoma	20	11	25~63	51.6
Nodular goiter	15	9	43~71	56.4

### MRI

Imaging was carried out on a 1.5 T MR (Signa Excite HD Twinspeed, GE Healthcare, USA) using a four-channel array coil, by conventional MRI sequences and transverse single shot echo-planar DWI (b values, 0 and 400 s/mm^2^). The main parameters of MRI sequences are presented in Table 2.

**Table 2:** Main scan parameters and sequences for thyroid gland evaluation

MR Imaging Sequence	TR (ms)	TE (ms)	FOV (mm^2^)	Matrix	Slice Thickness(mm)	Spacing (mm)	NEX	Number of Slices
Axial T_1_WI	520	14	200 × 200	320 × 192	4	0.5	3	12~14
Axial T_2_WI	3500	102	200 × 200	320 × 224	4	0.5	4	12~14
Coronal T_1_WI	550	11.1	240 × 240	320 × 224	3	0.5	4	12~14
Coronal T_2_WI	3000	102	240 × 240	320 × 224	3	0.5	4	12~14
Axial DWI (b = 0, 400 s/mm^2^)	6000	60.1	200 × 200	128 × 128	4	0.5	4	12~14

### Data analysis

Inclusion and exclusion of patients were carried out by the same senior radiologist (10-year experience in head and neck radiology). The reader was not aware of histopathological findings. DWI images were analysed based on the lesion’s signal intensity relative to adjacent noncancerous thyroid tissues on axial T_1_WI and T_2_WI.

DWI data and ADC maps were analysed with the AW 4.3 software (GE Healthcare). Two senior radiologists (10- and 8-year experience in head and neck radiology, respectively) independently measured the lesions’ ADCs according to three ROI methods: placement of three circular ROIs (small solid-sample method); placement of a freehand ROI outlining the tumor on a single slice (single-slice method); and placement of freehand ROIs outlining the tumor on each slice containing the tumor (whole-volume method).

In the small solid-sample technique, average ADC was obtained from 3 circular ROIs within the tumor regions, with the highest cellular activity on DWI of b_400_ (median ROI area, 22 mm^2^; range, 18–26 mm^2^). In the single-slice technique, the ROI was defined by tracing a line along the perceived tumor margins on DWI of b_400_ (median ROI area, 217 mm^2^; range, 33–970 mm^2^). In the whole-volume technique, ROIs were made along the perceived tumor borders on DWI of b_400_, covering the whole tumor region on every tumor-containing slice (median ROI area, 896 mm^2^; range, 51–5201 mm^2^), and ADC values in all sections were averaged for further analysis.

### Statistical analysis

Statistical analyses were carried out with the Medcalc software (Version 13.0.0.0, MedCalc software). Interobserver variability of tumor ADCs in all ROI methods was assessed by determining interclass correlation coefficients (ICCs), with the values of 0–0.20, 0.21–0.40, 0.41–0.60, 0.61–0.80 and 0.81–1.00 reflecting poor, fair, moderate, good and excellent correlations, respectively [23] as previously proposed [24]. Average ADCs in various ROI selection techniques were compared by the Friedman test, with post-hoc assessment by the Wilcoxon signed-rank test [25]. Statistical significance thresholds for the Friedman and post-hoc tests were set at *P <* 0.05 and *P <* 0.017 (0.05/3), respectively [21].

## Results

### Interobserver ADC variability

**Fig. 1:**
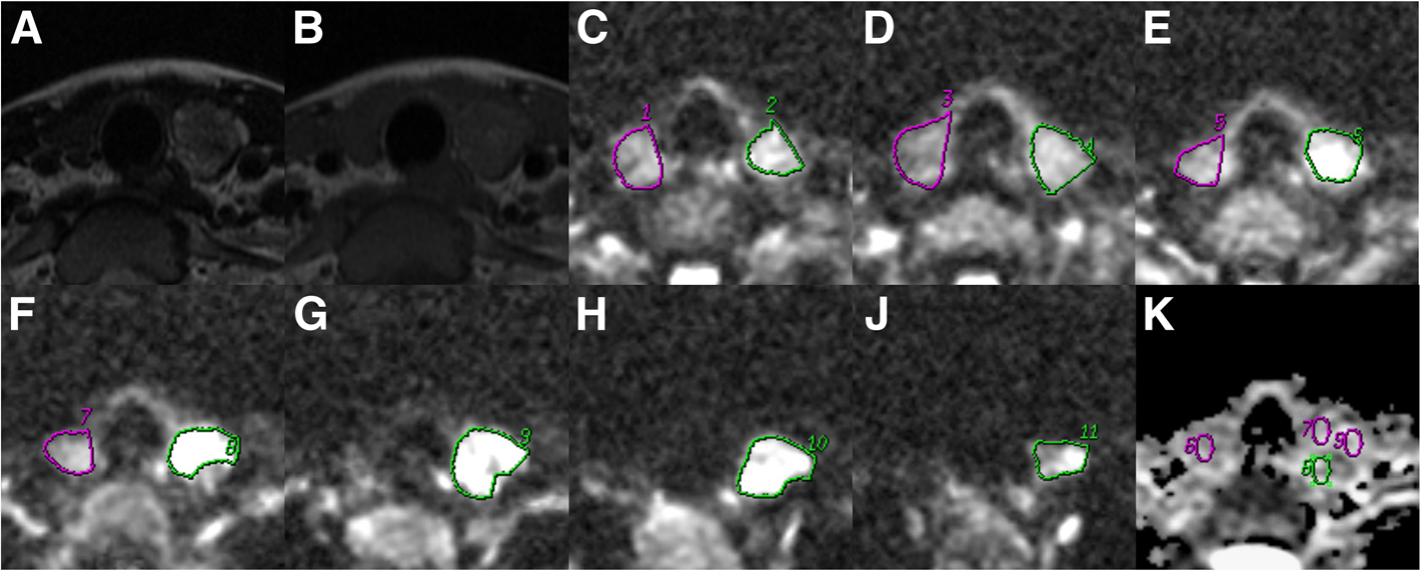
Images obtained from a 28-year-old female patient with histologically proven follicular thyroid carcinoma in the left lobe. In the whole-volume (**c**-**j**) and single-slice (**g**) methods, freehand ROIs were drawn along the high signal intensity border of the tumor to cover the entire tumor on DWI at b_400_. In the solid sample method, three circular ROIs were placed within the tumor areas of the highest cellular activity on DWI at b_400_. The tumor areas showed high signals on DWI at b_400_ and low signals on the ADC map (**k**). **a**, Axial T_2_WI; **b**, Axial T_1_WI; **c**-**j**, DWI (b_400_); K, ADC map

Axial DWI data and ADC maps for ADCs obtained with the various ROI selection techniques are depicted in Fig. 1. Average ADCs for benign and malignant thyroid nodules assessed by two readers employing various ROI selection techniques are presented in Tables 3 and 4, respectively.

**Table 3:** Inter-observer comparison of apparent diffusion coefficient (ADC) values (× 10^− 3^ mm^2^/s) for benign thyroid nodules obtained by the whole-volume, single-slice and solid-sample ROI methods by two independent readers

Parameters	Solid-sample ROI		Single-slice ROI		Whole-volume ROI	
	Reader 1	Reader 2	Reader 1	Reader 2	Reader 1	Reader 2
ADC_400_	2.03 (1.88, 2.27) [1.48, 2.93]	2.02 (1.96, 2.20) [1.67, 2.95]	2.03 (1.91, 2.36) [1.73, 3.17]	2.06 (1.93, 2.18) [1.65, 3.24]	2.05 (1.94, 2.28) [1.80, 3.25]	2.06 (1.92, 2.19) [1.73, 3.26]

*Data are expressed as median; numbers in parentheses are first (q1) and third (q3) quartiles; numbers in brackets are ranges

**Table 4:** Inter-observer comparisons of apparent diffusion coefficient (ADC) values (× 10^− 3^ mm^2^/s) for malignant thyroid nodules obtained by the whole-volume, single-slice and solid-sample ROI methods by two independent readers

Parameters	Solid-sample ROI		Single-slice ROI		Whole-volume ROI	
	Reader 1	Reader 2	Reader 1	Reader 2	Reader 1	Reader 2
ADC_400_	1.67 (1.47, 1.79) [1.00, 2.01]	1.63 (1.53, 1.88) [1.22, 2.01]	1.84 (1.65, 1.90) [1.25, 2.07]	1.74 (1.63, 1.87) [1.35, 2.15]	1.70 (1.57, 1.81) [1.23, 1.96]	1.78 (1.61, 1.85) [1.33, 2.04]

*Data are expressed as median; numbers in parentheses are first (q1) and third (q3) quartiles; numbers in brackets are ranges

**Fig. 2:**
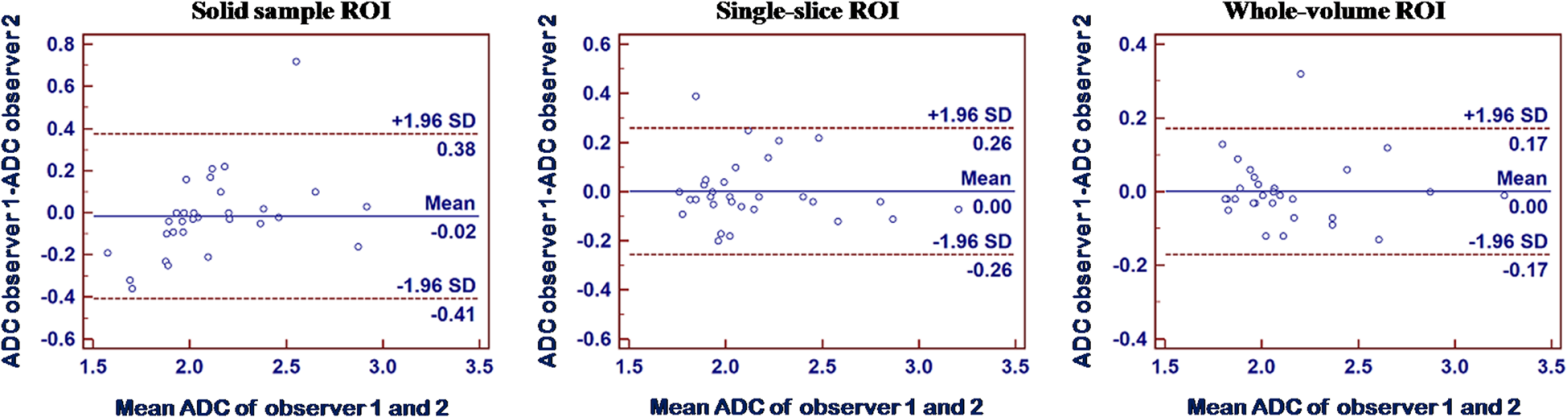
Interobserver reproducibility of mean ADC measurements (×10^−3^ mm^2^/s) for all three ROI methods in benign thyroid nodules. In Bland-Altman plots, the differences in mean ADC measurements (y-axis) were plotted against the mean ADCs (x-axis). Continuous line, mean absolute difference (bias); dashed line, 95% confidence interval of the mean difference (limits of agreement)

For ADCs in benign thyroid nodules, average differences and limits of agreement (LOAs) between readers were 0.00 [− 0.17–0.17] × 10^− 3^ mm^2^/s for the whole-volume group (ICC = 0.967), 0.00 [− 0.26–0.26] × 10^− 3^ mm^2^/s for the single-slice technique (ICC = 0.932) and − 0.02 [− 0.38–0.41] × 10^− 3^ mm^2^/s for the small solid-sample group (ICC = 0.823), as shown in Fig. 2.

**Fig. 3:**
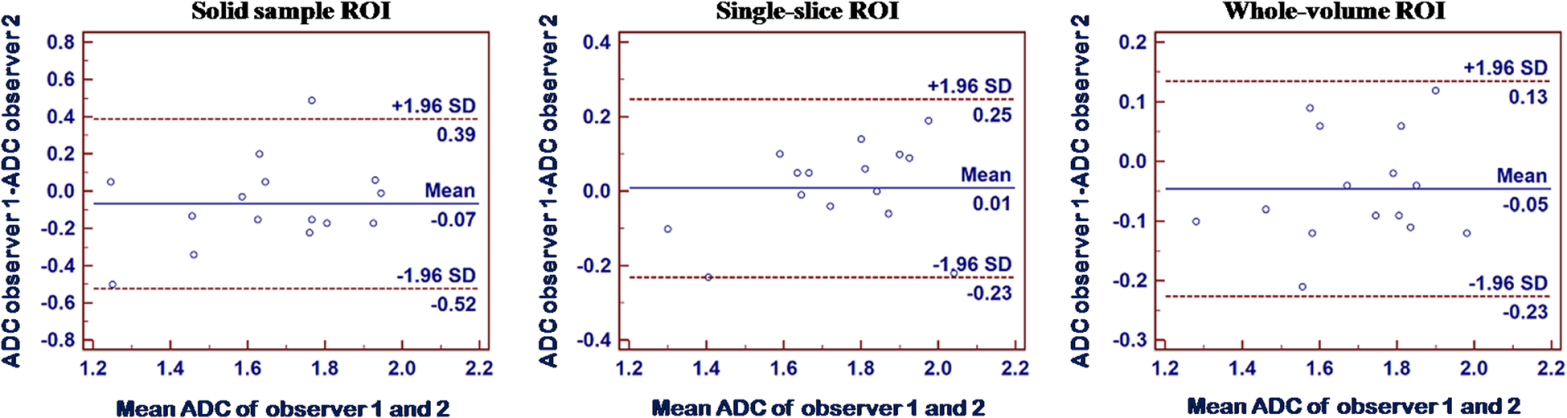
Interobserver reproducibility of mean ADC measurements (× 10^− 3^ mm^2^/s) for all three ROI methods in malignant thyroid nodules. In Bland-Altman plots, the differences in mean ADC measurements (y-axis) were plotted against the mean ADCs (x-axis). Continuous line, mean absolute difference (bias); dashed line, 95% confidence interval of the mean difference (limits of agreement)

For ADCs in malignant thyroid nodules, average differences and LOAs between readers were − 0.05 [− 0.23–0.13] × 10^− 3^ mm^2^/s for the whole-volume group (ICC = 0.885), 0.01 [− 0.23–0.25] × 10^− 3^ mm^2^/s for the single-slice technique (ICC = 0.839) and − 0.07 [− 0.52–0.39] × 10^− 3^ mm^2^/s for the small solid-sample group (ICC = 0.579), as shown in Fig. 3.

### ADCs in the three ROI selection techniques

**Fig. 4:**
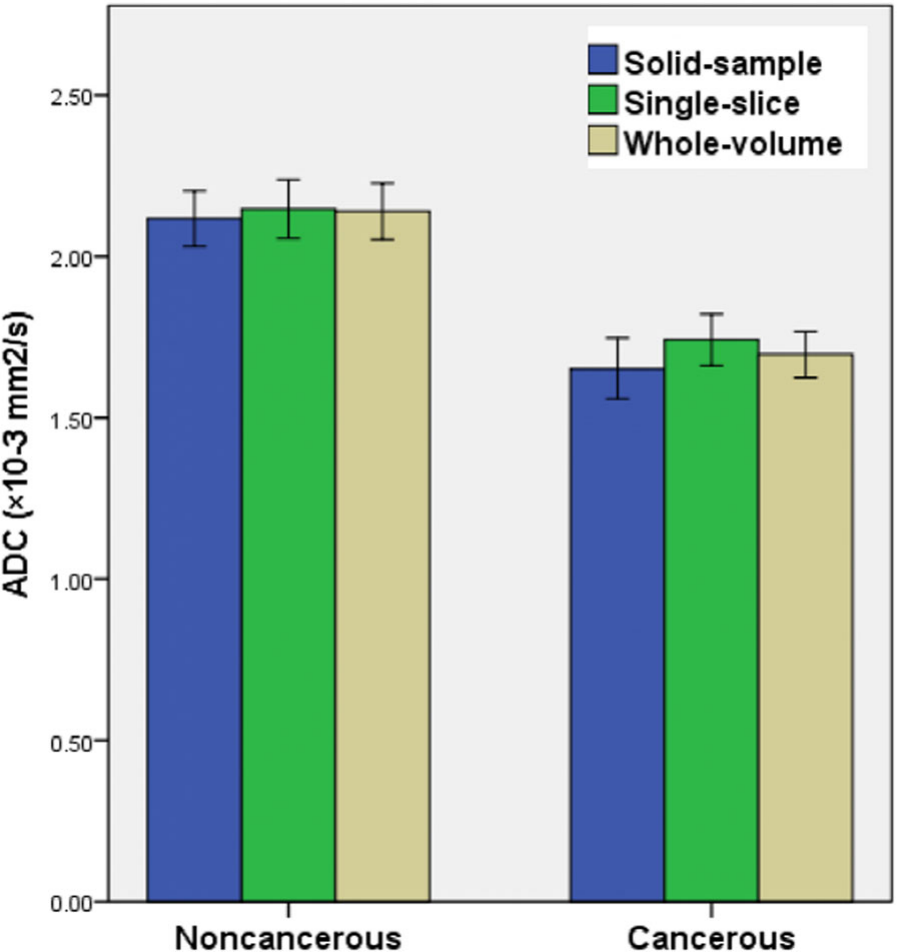
Mean ADC (× 10^− 3^ mm^2^/s) measured by the whole-volume, single-slice and solid-sample methods for noncancerous and cancerous thyroid nodules

Average ADCs for noncancerous and cancerous thyroid nodules obtained by the three ROI selection techniques are summarized in Table 5 and Fig. 4, respectively. The mean ADCs of b_400_ were more scattered in the small solid-sample group in comparison with the other two techniques for both noncancerous and cancerous thyroid nodules. The Friedman test demonstrated no significant differences in ADCs for noncancerous thyroid nodules among the various techniques (*P* = 0.797), while cancerous specimens had significant differences (*P* < 0.001).

**Table 5:** Comparisons of mean ADC_400_ (× 10^− 3^ mm^2^/s) (± standard deviation, SD) measured by the whole-volume, single-slice and solid-sample methods for noncancerous and cancerous thyroid nodules

Category	Solid-sample ROI	Single-slice ROI	Whole-volume ROI	*P*
Noncancerous thyroid nodules	2.12 ± 0.32	2.15 ± 0.35	2.14 ± 0.34	**0.797**
Cancerous thyroid nodules	1.65 ± 0.22	1.74 ± 0.21	1.70 ± 0.19	**< 0.001**

### Diagnostic performances of the three ROI selection techniques in noncancerous and cancerous nodules

**Fig. 5:**
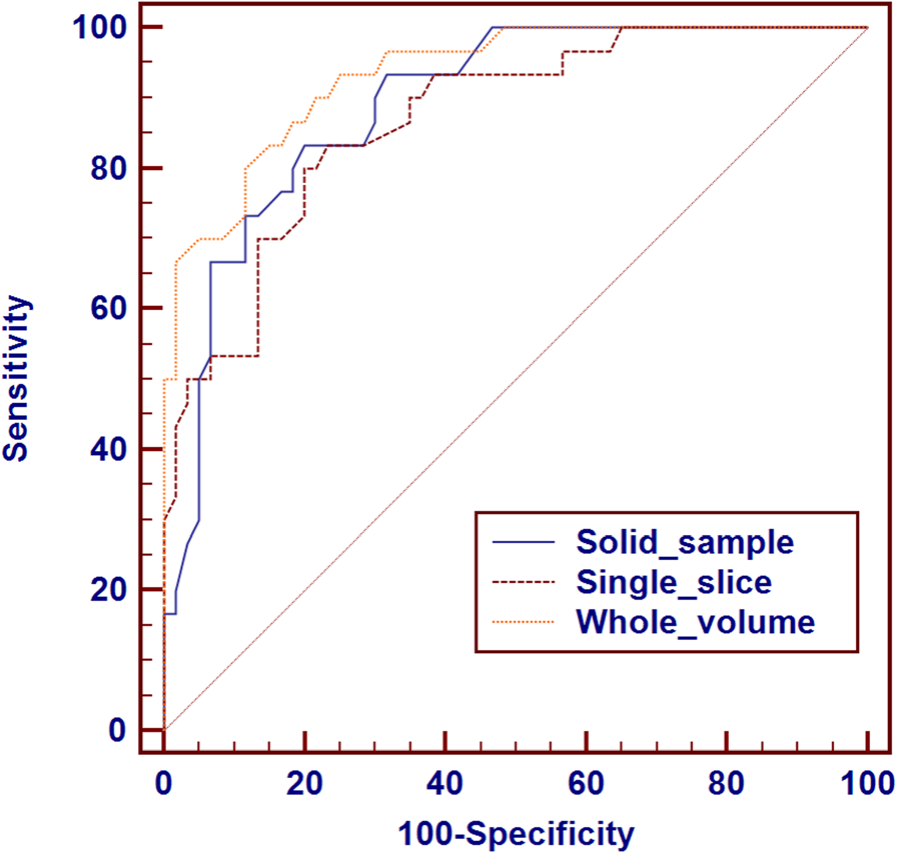
ROC curves of ADC values measured by the whole-volume, single-slice and solid-sample methods for benign and malignant thyroid nodules

Compared with benign nodules, the areas under the ROC curves (AUC values) used to identify malignant nodules according to the ADCs obtained in the three ROI methods were 0.891, 0.870, and 0.934, respectively. Among the three ROI selection methods, the whole-volume method-derived ADC had the highest combined sensitivity (80.0%), specificity (88.3%) and Youden index (0.683), with 1.84 × 10^− 3^ mm^2^/s as cut-off. ROC data for the diagnostic performances of the three ROI techniques in differentiating noncancerous and cancerous nodules are depicted in Fig. 5 and Table 6.

**Table 6:** ROC analyses of ADC measured by the whole volume, single slice and small solid-sample methods for benign and malignant thyroid nodules

Category	Small solid-sample ROI	Single-slice ROI	Whole-volume ROI
AUC	0.891	0.870	0.934
Cut-off value (×10^−3^ mm^2^/s)	1.90	1.90	1.84
Sensitivity	83.3%	83.3%	80.0%
Specificity	80.0%	78.3%	88.3%
Youden Index	0.616	0.600	0.683

## Discussion

Our results demonstrated that the reproducibility of ADC measurements of both noncancerous and cancerous thyroid nodules in the whole-volume and single-slice methods was acceptable, with average interobserver bias not exceeding 0.1 × 10^− 3^ mm^2^/s and LOAs below 0.3 × 10^− 3^ mm^2^/s [21]. The reproducibility of average ADC measurements of both noncancerous and cancerous thyroid nodules in the solid-sample ROI selection method was inadequate because of the scattered mean ADCs in Bland-Altman test results, with the limits of agreement over ±0.30 × 10–3 mm2/s for the small solid-sample group. Mean ADC measurements have been employed to assess the ADC diagnostic threshold in distinguishing noncancerous thyroid nodules from cancerous ones in previous studies [1–20]. However, the reliability of ADC data obtained with the above ROI selection techniques in thyroid nodules has been rarely evaluated. Previously, the effects of the three ROI selection techniques on tumor ADC and interobserver variability in patients with pancreatic [21] and advanced colorectal [22] cancers have been assessed. The latter reports demonstrated that ROI size and placement considerably impact the tumor’s mean ADC as well as interobserver variability [21, 22], with the whole-volume technique showing the highest reproducibility [21, 22]. A consensus report by Padhani et al. advocated that basic standards for tissue diffusion coefficient assessment and reporting are necessary, and strongly warned against the use of small delineations in ADC measurements [26]. Therefore, a feasible method should be determined to standardize ADC measurements in thyroid nodules. Additionally, we found that the whole-volume method for ROI selection yielded the highest diagnostic value for differentiating noncancerous and cancerous thyroid nodules.

The reproducibility of average ADC measurements for both noncancerous and cancerous thyroid nodules in the solid-sample ROI selection method was inadequate because of the scattered mean ADCs in the Bland-Altman test. In the current study, the solid-sample technique was limited to the size and position in the tumor area, significantly varying from one patient to another, which could be due to pathological and structural differences. In clinic, the solid-sample technique is commonly employed to obtain the ADCs of thyroid nodules [1–20]. Here, three circular ROIs were placed within solid tumor areas showing high signals on DWI. Two investigators independently measured tumor ADCs using the solid-sample method, without consensual selection of the same slice and position for the ROI in each case. Tumors usually show heterogeneity, especially in cases with thyroid goitre and adenoma, and ROIs should be placed away from cystic areas. In this study, the solid-sample method yielded the worst interobserver variability for mean ADC measurements. Therefore, the solid-sample method derived ROI is not optimal for ADC measurements of thyroid nodules.

Regarding benign thyroid nodules, obvious differences were observed in mean ADCs among the assessed ROI selection techniques. In ADC measurement of malignant thyroid nodules by the small solid-sample technique derived ROI, the most viable solid tumor portions were ncluded in the ROI, while blood vessels as well as the tumor border and cystic parts were excluded. Blood vessels or the tumor boundary could elevate the ADCs due to the tissue having high blood perfusion along the vessel or boundary, which accelerates the diffusional movement of water molecules. In the whole-volume and single-slice methods, blood vessels and the tumor border were easily included in the ROI, which may lead to relatively elevated ADCs. Furthermore, previous studies reported markedly decreased ADCs in cancerous nodules but not in noncancerous and normal tissues [8–12], corroborating the current work. The solid portions of malignant thyroid nodules contain areas with hypercellularity and high nucleocytoplasmic ratio, resulting in reduced extracellular space and limited cellular diffusion, which may lead to low ADC values [15]. Additionally, field strength, b-value selection and the post-processing approach employed potentially contribute to the final ADC [27]. As stated above, DWI was carried out on the same 1.5 T scanner in one hospital with the single-shot echo-planar imaging sequence to avoid instrument bias [1–12]. Some studies have used b values of 300, 400 and 500 s/mm^2^, respectively, for DWI of thyroid nodules, which can reduce the effects of blood perfusion to reflect the actual diffusion in the tissue [15–17]. Other studies have used higher b values (over 600 s/mm^2^) for DWI of thyroid nodules, which may increase susceptibility artefacts in DWI [2, 12]. The b value of 400 s/mm^2^ used in this study was suitable for the reproducibility of ADC measurements, and selected according to the signal-to-noise balance and DWI image quality [28]. In addition, a meta-analysis of DWI value in distinguishing cancerous thyroid nodules from noncancerous ones advocated for higher b values to increase diagnostic accuracy, although no notable differences in AUC values were found between the low and high b value groups [29].

The limitations of this work should be mentioned. Firstly, the b value for DWI in this study was low. DWI examinations were carried out with a b value of 400 s/mm^2^ to minimize susceptibility artefacts and ameliorate the SNR of the thyroid gland, while greater b values might show higher sensitivity and reflect the actual diffusion [29–31]. Additional b values for DWI should be assessed in further research, identifying the optimal b value for the detection of thyroid gland lesions. Secondly, the effects of ROI selection techniques on ADC measurement in distinct field strengths or b-values were not evaluated, which deserves further investigation.

## Conclusions

ROI selection overtly affects ADC and interobserver variability in thyroid nodules. Among the three ROI selection methods assessed, the small solid-sample technique showed highest interobserver variability for average ADC.

## Data Availability

All data related to this work are available from the corresponding author upon reasonable request.

## Abbreviations

ADC: Apparent diffusion coefficient
DWI: Diffusion-weighted imaging
FOV: Field of view
ICC: Interclass correlation coefficient of variance
LOA: Limit of agreement
MRI: Magnetic resonance imaging
ROI: Region of interest
T_1_WI: T_1_-weighted imaging
T_2_WI: T_2_-weighted imaging
